# Deciphering the Molecular Profile of Lung Cancer: New Strategies for the Early Detection and Prognostic Stratification

**DOI:** 10.3390/jcm8010108

**Published:** 2019-01-17

**Authors:** Elisa Dama, Valentina Melocchi, Tommaso Colangelo, Roberto Cuttano, Fabrizio Bianchi

**Affiliations:** Fondazione IRCCS Casa Sollievo della Sofferenza, Unit of Oncology Biomarkers, 71013 San Giovanni Rotondo (FG), Italy; e.dama@operapadrepio.it (E.D.); v.melocchi@operapadrepio.it (V.M.); t.colangelo@operapadrepio.it (T.C.); r.cuttano@operapadrepio.it (R.C.)

**Keywords:** lung cancer, early diagnosis, prognosis, chemotherapy response, biomarkers, microRNA, gene expression, exosomes

## Abstract

Recent advances in radiological imaging and genomic analysis are profoundly changing the way to manage lung cancer patients. Screening programs which couple lung cancer risk prediction models and low-dose computed tomography (LDCT) recently showed their effectiveness in the early diagnosis of lung tumors. In addition, the emerging field of radiomics is revolutionizing the approach to handle medical images, i.e., from a “simple” visual inspection to a high-throughput analysis of hundreds of quantitative features of images which can predict prognosis and therapy response. Yet, with the advent of next-generation sequencing (NGS) and the establishment of large genomic consortia, the whole mutational and transcriptomic profile of lung cancer has been unveiled and made publicly available via web services interfaces. This has tremendously accelerated the discovery of actionable mutations, as well as the identification of cancer biomarkers, which are pivotal for development of personalized targeted therapies. In this review, we will describe recent advances in cancer biomarkers discovery for early diagnosis, prognosis, and prediction of chemotherapy response.

## 1. Circulating Biomarkers for Lung Cancer Early Diagnosis

Lung cancer causes ~1.7 million deaths every year due to frequent late diagnoses when the disease is metastatic [1]. Primary prevention strategies, such as anti-smoking campaigns as well as screening programs, represent the gold standard to limit lung cancer burden and reduce mortality. After the first studies failed to demonstrate a reduction in lung cancer specific mortality by the sole use of chest X-ray, low-dose computed tomography (LDCT) was then introduced as an effective diagnostic tool to augment early diagnoses. First encouraging results came in 2011, when the United States National Lung Screening Trial reported a 20% reduction in lung cancer-specific mortality mainly due to a higher sensitivity of LDCT vs. chest X-ray. However, the application of LDCT in screening program is currently controversial due to the (i) high false positive rate, (ii) uncertainty about the optimal interval length of screening rounds, and (iii) cost-effectiveness of the LDCT screening protocol in high-risk individuals [1]. In order to improve the cost-effectiveness ratio, several comparative studies of the different criteria for the selection of at-risk populations (i.e., individuals which will largely benefit from the screening) were performed [2,3]. Beside the use of standard eligibility criteria based on age and smoking exposure, the combination of additional risk factors in accurate epidemiological risk models, such as gender, race, ethnicity, education, body mass index, previous or existing lung disease, exposures to dust or asbestos, and personal family history of lung cancer, outperform the eligibility criteria used in previous screening trials [4,5]. Furthermore, minimally invasive strategies, based on radiomics and/or circulating biomarkers, are promising and can complement LDCT to augment the overall performance of screening protocols for lung cancer early detection. Fully automated techniques to extract and quantify features from radiation images were investigated for many years, and the correlation between the captured structures and malignancy of nodules was demonstrated [6,7]. Nowadays, radiomics researchers are focused on the combination of both quantification and visual assessment, to provide more comprehensive information on imaging databases and manage the lack of reproducibility. Moreover, the combination of imaging and molecular information (e.g., mutational and expression profile) led to the development of radiogenomics signatures which were exploited to predict the prognosis of lung cancer patients [8].

A large set of data also support the efficacy of circulating biomarkers as minimally invasive diagnostics [9,10]. Circulating tumor cells, circulating cell-free nucleic acids (cf-RNA and cf-DNA), and circulating proteins are still under investigation and not yet fully incorporated into routine clinical practice. Circulating cell-free microRNAs (cf-miRNAs) represent the most promising and valuable class of non-invasive molecular biomarkers for the detection of several cancers, including lung cancer. MicroRNAs are a group of short (~22 nucleotides long) non-coding single stranded RNAs that regulate gene expression post-transcriptionally. A single miRNA can target hundreds of mRNAs, thus regulating the expression of a large number of genes. miRNA are frequently altered in cancer and were shown to regulate cancer phenotypes, including proliferation, cell migration, and apoptosis [11]. MicroRNA are the most abundant cf-RNA molecules in the blood, and showed remarkable long-term stability in plasma and serum samples [12], making them useful biomarkers for cancer detection. Several studies proposed a variety of cf-miRNA signatures diagnostic for lung cancer (Table 1). For example, Yang et al. meta-analyzed a multitude of studies (*N* = 134) where cf-miRNA profiles were explored, for a total of 6919 patients with lung cancer and 7064 controls, including serum, plasma, peripheral blood mononuclear cells/neutrophils, and peripheral blood [13]. The pooled data showed high diagnostic accuracy for detecting lung cancer patients with a sensitivity of 0.83 (95% CI, 0.80–0.85), specificity of 0.84 (0.82–0.86), and corresponding AUC of 0.90 (0.88–0.93). A valuable aspect of this meta-analysis involves the information provided by subgroup analysis, such as number of cf-miRNA predictor, specimen type, source of control, and histology type and stage, which further supported the evidence for a relevant diagnostic role of cf-miRNA. 

However, very few cf-miRNA-based biomarkers were derived and validated in the actual screening population (Table 1), which is mandatory to show the performance of these biomarkers for the diagnoses of small, localized, and asymptomatic malignant lung nodules. Montani et al. validated a 13 serum cf-miRNA panel in a large cohort (*N* = 1115) of high-risk individuals (>20 pack-year smoking history, aged >50 years) enrolled in the COSMOS screening trial, showing a sensitivity of 0.78, specificity of 0.75, and an AUC of 0.85 [14]. Sozzi et al. validated a plasma cf-miRNA signature from patients in the MILD screening study, including 939 participants with 0.87 of sensitivity and 0.81 of specificity (Table 1) [15]. Although cf-miRNAs were largely investigated as potential diagnostic markers, the low overlap and the lack of standardization of screening platforms [16,17] is actually limiting the application of miRNA screening in the clinical routine. For example, the limited sample sizes, heterogeneous criteria for selection of cases and controls, and lack of external validation may contribute to unreliability of selected biomarkers [18]. Moreover, there is a high variability in the cf-miRNA quantification when different platforms (i.e., microarray, qPCR, and RNA-seq) were compared [19]. Lastly, the variation in pre-analytical factors, such as patient fasting, hemolysis, RNA isolation protocol [20], references for data normalization [21], together with different statistical approaches used for cf-miRNA profiles, make the comparison between studies difficult, and could somewhat explain the lack of reproducibility of results [22].

**Figure 1:**
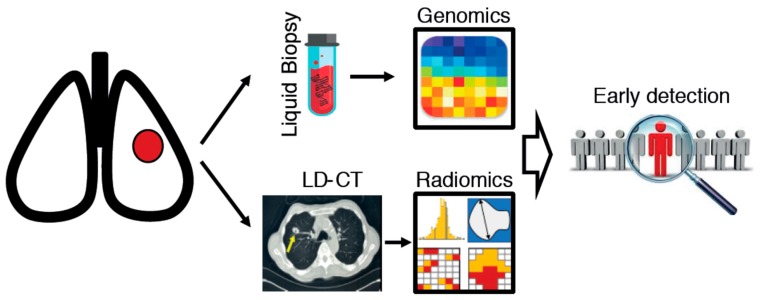
Schematic representation of an integrated analysis of circulating biomarkers (liquid biopsy) and radiomics, to improve lung cancer early diagnosis in at-risk individuals (defined by epidemiological risk models).

Future studies combining epidemiological factors, radiogenomics signatures, and biomarkers are expected to improve risk prediction models for the early diagnosis of lung cancer (Figure 1). Moreover, the assessment of the clinical consequences of classification of individuals into a risk group is also required. A method borrowed from utility theory—decision curve analysis—was proposed as a suitable approach to measure the clinical value of a prediction model, beyond what common statistical measures may suggest, e.g., the area under the curve and net reclassification index [23]. The results of a decision curve analysis can be easily translated in clinically applicable terms, since it evaluates the benefits and harms of diagnosing patients with disease versus unnecessary additional testing for healthy individuals. The application of such an approach could complement refined risk prediction models for early diagnosis of lung cancer.

## 2. Cancer Biomarkers and Exosomes

**Figure 2:**
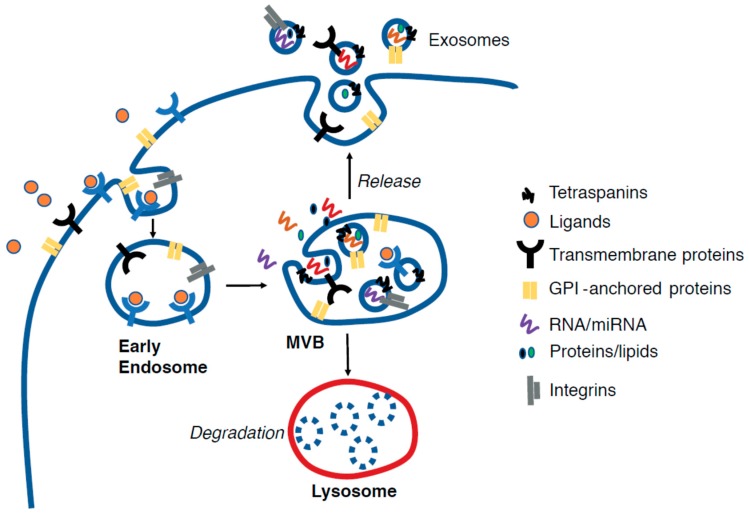
Schematic representation of exosomes biogenesis. The in-budding of endosomes originates the multivesicular bodies (MVBs) which can follow different fates: (i) fusion with the lysosome, which leads to degradation of MVB content; (ii) fusion with the cell membrane and release of intraluminal vesicles to the extracellular space, thus generating exosomes. The exosomes are enriched with several proteins located on the membrane including, but not limited to, transmembrane proteins/receptors, integrins, glycophosphatidylinositol (GPI)-anchored proteins, and tetraspanins, which are specific markers of exosomes. Intra-exosomal proteins, nucleic acids (coding and non-coding RNA), lipids, and other metabolites, do also exist, and are loaded during the invagination of MVB membranes. Such molecules can be transferred to host cells once exosomes are released by donor cells and activate signaling pathways.

Virtually all circulating biomarkers are present in extracellular microvesicles named exosomes. Exosomes (EX) are cell-derived vesicles of nanoscale size (30–120 nm) originating from the in-budding of endosomes that, in turn, generates multivesicular bodies (MVBs) which, by invagination of the MVB membrane, form intraluminal vesicles (ILVs) (Figure 2). Upon fusion of MVBs to cell membrane, the ILVs are released in the extracellular space, thus becoming mature exosomes. EX contains proteins, nucleic acids (RNA, miRNA, mtDNA, DNA), lipids, and other metabolites [31,32] which were loaded in EX when resident in the MVB through the action of ALIX (ALG-2-interacting protein X) and of the endosomal sorting complex protein complex (ESCRT). These cargos can be transferred from one cell to another, thus permitting molecular communication. 

Recent reports described that tumor-released exosomes (TEX) are “decorated” with cancer-related molecules [33,34,35,36], which were shown to influence tumor development by stimulating oncogenic pathways activation, conferring chemoresistance, favoring immuno-escaping, reprogramming tumor microenvironment, and preparing pre-metastatic niche [37,38,39,40]. Importantly, the overall amount of EX appeared to be higher in lung cancer patients. Rabinowits et al. [41] provided initial indications of the diagnostic value of measuring EX in blood samples since they observed that mean exosome concentration was 3-fold higher (2.85 mg/mL vs. 0.77 mg/mL) in the blood from patients with lung cancer vs. healthy subjects (*P* < 0.001). Similar evidence was also found in an analysis of EX concentration in bronchoalveolar lavage samples, which resulted in being higher in lung tumor patients compared to normal samples [42]. Importantly, the expression level of EGFR was significantly increased in TEX from lung cancer patients with respect to EX from normal individuals, which suggests that the measurement of TEX protein levels can be diagnostic for lung cancer [43]. In keeping with this idea, a novel approach combining surface-enhanced Raman spectroscopy (SERS) with principal component analysis (PCA) identified a specific surface pattern of proteins and lipids on lung TEX with a sensitivity and specificity of 0.95 and 0.97, respectively [44]. Likewise, a differential abundance of cell signaling proteins (e.g., EGFR, GRB2, and SRC) was identified in lung TEX using a triple SILAC quantitative proteomic analysis [45]. 

Further studies were carried out which allowed for discovering mRNA, non-coding RNA (e.g., microRNA and lncRNA), circular RNA (circRNA) [46], and DNA [47], including the detection of actionable mutations, such as the T790M mutation in EGFR, in exosomal (Exo)-DNA and Exo-RNA [48]. Remarkably, a sizable fraction of microRNAs was found in exosomes, and was diagnostic for lung cancer. For example, Rabinowits et al. identified 12 specific exosomal miRNAs (Exo-miRNA) biomarkers for lung adenocarcinoma [41] while, in another study, two miRNA exosome plasma tests were developed to diagnose lung cancer (Exo-miR-200b-5p/378a/139-5p/379) and also to discriminate granuloma from lung adenocarcinoma (Exo-miR-629/30a-3p/100/200b-5p/154-3p/151a-5p) [49]. Several other Exo-miRNAs were recently described as being diagnostic for lung cancer [50,51]. An updated list can be found in Table 2.

Besides, Zhang et al. showed the opportunity of analyzing long non-coding RNA (lncRNA) in exosomes for the diagnosis and prognosis of non-small cell lung cancer (NSCLC). In particular, lncRNA *MALAT-1* was found to be highly expressed in NSCLC patients and correlate with tumor progression [52]. Interestingly, Li et al. have reported that lung TEX contains circular RNAs (circRNAs) (Table 2) [53] which were found enriched compared to the intracellular level [53]. Circular RNAs were originally thought to be a byproduct of aberrant splicing without a precise biological function. However, new evidence is now raising indicating their possible role as miRNA sponges, thus with the potential to modulate miRNA-regulatory functions [54]. It is worth mentioning that EX can be exploited to deliver anticancer drugs and target single cells [55]. Definitely, their nanoscale size facilitates diffusion within tissues, including the blood–brain barrier [56]. Discovery of TEX cargo has drawn increasing attention due to the exerted effect on oncogenic pathways activation, conferring chemoresistance, favoring immuno-escaping, reprogramming the tumor microenvironment, and preparing the pre-metastatic niche [33,37,38,57]. Overall, the analysis of EX in the blood can be functional, either for biomarker discovery to anticipate the diagnosis of cancer, or even for targeting the hallmarks of cancer at single-cell resolution.

## 3. Cancer Biomarkers and Molecular Subtyping of Lung Cancer

Despite these efforts to anticipate the diagnosis of lung cancer, a significant fraction of patients with stage I disease (~20%) will continue experience to tumor recurrence and metastases [63]. This is principally due to a high level of genetic heterogeneity of this disease, which impinges on tumor evolution and metastatic dissemination [64,65]. However, recent advances in high-throughput genomic screening allowed a more in-depth characterization of molecular features of lung cancer, with the aim of deconvoluting heterogeneity and developing prognostic biomarkers. For instance, Wistuba et al. reported an mRNA expression signature of cell-cycle progression (CCP) genes that predicts cancer-related death from early stage lung cancer [66]. Wilkerson et al. proposed a signature of ~500 genes able to stratify patients with lung adenocarcinoma into three molecular subtypes, with distinct and peculiar molecular characteristics [67]. Further analysis of these subtypes was performed by the Cancer Genome Atlas consortium (TCGA) which integrates gene expression, methylation, somatic mutations, copy number variations (CNV), and proteomic profiles of a large cohort of lung adenocarcinomas [68]. The TCGA study proposed a new nomenclature for lung cancer subtypes which integrated the transcriptional subtypes with the histopathological, anatomic, and mutational categorizations, i.e., the terminal respiratory unit (TRU, formerly bronchioid); the proximal-inflammatory (PI, formerly squamoid); and the proximal-proliferative (PP, formerly magnoid). These three subtypes are characterized by specific genetic alterations: the PP subtype is enriched for mutation of KRAS oncogene and inactivation of the STK11 tumor suppressor gene; the PI subtype was characterized by mutations in NF1 and TP53 genes; the TRU subtype frequently carried mutations in the EGFR gene, as well as tumors expressing the kinase fusion. In addition, among these three different subtypes, there was a variability in terms of mutation rate, frequencies of transition mutations, genomic ploidy, large-scale genomic aberrations, smoking history, and survival. Furthermore, the existence of an aggressive lung cancer molecular subtype (C1 subtype) in early stage lung cancer (stage I) has recently been described [69]. The C1 subtype has a peculiar gene/protein expression and mutational profile, resembling advanced metastatic cancer. Of note, inactivating mutations in KEAP1 gene is a hallmark of this C1 aggressive subtype [69,70]. KEAP1 is an E3 ligase that target Nrf2 protein (i.e., a redox-sensitive transcription factor) to 26S proteasome. Nrf2 participate to oxidative stress response through activation of genes encoding antioxidants, xenobiotic detoxification enzymes, and drug efflux pumps [71,72]. Nrf2 is actually under study as a potential therapeutic target in a variety of clinical trials including different type of tumor disease and advanced stage IV lung cancer (ClinicalTrials.gov Identifier: NCT02417701).

Recent studies have also brought to attention the relevance of characterizing tumor-infiltrating immune cells. Kargl et al. performed a comprehensive analysis of 51 immune cells population in a cohort of NSCLC tissues [73] which revealed that ~50% of immune cells were of myeloid origin, and neutrophils were the most abundant immune cell population. Furthermore, specific subsets of tumor-infiltrating lymphocytes (TIL) were found enriched in lung cancer tissue when compared to normal adjacent tissues, such as B cells (CD19+ CD20+), T cells (CD3+), CD4+ cells, and CD8+ cells [73]. Therefore, the characterization of the amount of TIL and the kind of immune cells enriched can be relevant to better understandign lung cancer biology and to develop cancer biomarkers [74]. The variability of the amount and type of TILs contributes to the biological heterogeneity of the tumor and can determine its aggressiveness. Indeed, TIL was shown to correlate with prognosis in NSCLC, i.e., patients with a high TIL have a favorable prognosis, while patients with low TIL have an adverse prognosis [75]. Considering that most of the TIL is composed by T lymphocytes and that the CD8+ is the effector arm, tumors with a high TIL should be sensitive to adaptive immunity. However, cancer cells can activate immune checkpoints mechanisms (e.g., PD1, PD-L1, CTLA4) which maintain immune homeostasis by inhibiting autoimmunity [76]. In addition, it has been recently shown that while the increased mutational load would increase the formation of neoantigens whose exposure on the MHC-I complex elicit cytotoxic CD8+ and NK cells’ antitumor response, the intratumoral genetic variability (i.e., subpopulation of tumor cells with diverse mutation profile) would favor infiltration of macrophages (TAM), T regulatory cells (Treg), and myeloid-derived suppressor cells (MDSCs) which release pro-tumor factors, ultimately favoring a bad prognosis [77].

Several gene expression signatures specific for TIL were shown to be effective to deconvolute the different populations of TILs and predict the immune response behavior of cancer [78]. The use of “TIL signatures” as biomarkers to predict response to immune checkpoint inhibitors (i.e., anti-PD-1/PDL1, CTLA4) hold the potential to augment the efficacy of lung cancer therapy and, ultimately, increase lung cancer patients’ overall survival. 

## 4. Cancer Biomarkers Predictive of Lung Cancer Chemotherapy Response

**Figure 3:**
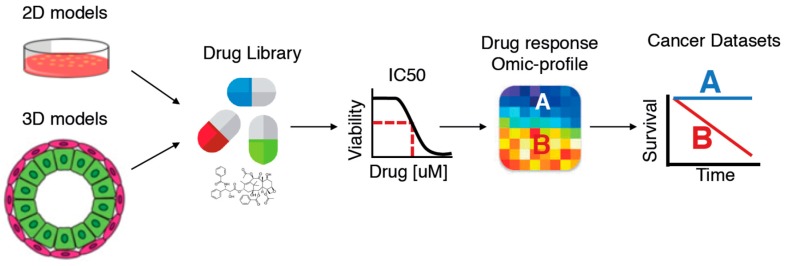
Drug library screening using 2D and 3D lung cancer models can unveil cancer biomarkers predictive of chemotherapy response. Cells are treated with different concentration of the tested compounds and the IC50 (i.e., the concentration at which cell proliferation was inhibited by 50%) is computed to determine chemoresponsivness of every cell line (i.e., sensitive vs. resistant). Next, a molecular profile analysis (e.g., gene expression, mutational, protein expression, methylation) can be performed in order to identify a signature correlating with chemoresponsivness, which can be eventually applied to cohorts of patients, to predict their response to treatment and/or to identify actionable pathways.

Currently, there is a lack of molecular predictors of chemotherapy response for lung cancer. In addition, response rates to chemotherapy and benefits in term of survival are also hindered by the development of drug resistance [79,80,81]. Tumors can be intrinsically resistant to chemotherapy or, alternatively, can become chemoresistant during treatment as a result of several adaptive responses [82]. Moreover, the acquisition of drug resistance can be the consequence of the selection of a pre-existing resistant subpopulation of cells in the primary tumor during chemotherapy treatment [82]. The combination of platinum compounds with third-generation anticancer agents, such as gemcitabine, paclitaxel, or vinorelbine (platinum-based doublet chemotherapy) is the standard chemotherapeutic treatment for both early and advanced stage NSCLC patients [83]. Depending on the type of drug, several molecular mechanisms through which cancer cells acquire drug resistance have been reported. For instance, platinum compounds, such as cisplatin or carboplatin, are DNA-alkylating agents that bind covalently to DNA and form DNA adducts, thereby compromising cellular processes, such as DNA replication and transcription [84]. Either a lower expression of drug membrane transporters or a higher activity of detoxification proteins may cause tumor resistance by reducing intracellular drug concentration. Alternatively, cancer cells can avoid cell death after DNA adduct formation due to an increased DNA repair capacity or increased tolerance to DNA damage [84,85]. To overcome drug resistance, current research efforts aim to identify reliable biomarkers which predict chemotherapy response to be used in combination with newly discovered immuno- or molecularly targeted-therapies. Predictive biomarkers hold the potential to select those patients that will benefit from a particular treatment, thus avoiding the unnecessary exposure of non-responder patients to toxic effects. Candidate biomarkers can be drug transporters, proteins involved in the activation/inactivation of the drugs or factors which play a role at any level of the drug mechanism of action, such as DNA repair or cell cycle regulation. Although many candidate predictive biomarkers have been already explored, none of them still reached the clinical practice [86]. Technological advancement in high-throughput “omics” techniques now offer the possibility to manipulate big data for cancer heterogeneity deconvolution to score reliable biomarkers for precision cancer medicine [87]. In keeping with this, large pharmacogenomic screening have been conducted using immortalized cancer cell line in vitro [88,89] (Figure 3), which generated chemosensitivity profile for most of the drug used in cancer therapy [90,91]. Albeit, the in vitro 2D experimental system does not take account of a possible contribution of tumor microenvironment in the acquisition of drug resistance [92], immortalized cancer cell lines have been recently demonstrated to recapitulate to a similar extent in most of the genetic alterations observed in matched primary tumors [93]. Moreover, a recent work by Lee et al. [94] highlighted the possibility to use non-immortalized patient tumor-derived cell lines (PDCs) in large pharmacogenomic screenings, with the main advantage of using experimental models most closely resembling the molecular profile of the original tumor. Interestingly, because of the relative short time of derivation (within 2–3 weeks of biopsy), PDCs can could be successfully used to predict drug response of matched patients in “real-time”. 

Finally, the integration of pharmacogenomic data with molecular profiling of cancer cell lines and tumor samples (mutation profile, DNA methylation, miRNA/mRNA, and protein expression profile) is essential for the development of accurate biomarkers and their application in clinical trials [95]. Besides in vitro 2D culture conditions, recent advancement in 3D culture technologies have allowed a more efficient isolation and culture of organoids directly from patient tumor tissue [96], thus enabling drug screening in ex vivo conditions (Figure 3). For instance, a recent analysis revealed that patient-derived organoids allow an accurate prediction of treatment response of gastrointestinal cancer patients [97]. Since it is conceivable that the percentage of NSCLC patients diagnosed at early stage will increase due to an improvement of lung cancer screening protocols, as we previously discussed, future efforts needs to be more focused on the development of predictive biomarkers to assist cancer treatment decision-making.

## 5. Concluding Remarks

Preclinical studies were indeed successful to identify cancer biomarkers for the early diagnosis, prognostic stratification and prediction of chemotherapy response. Such biomarkers will ultimately bolster the implementation of personalized therapies to ameliorate clinical management of cancer patients. However, pitfalls in the design of cancer biomarkers preclinical studies have certainly delayed their transfer to the clinical setting. For example, the high heterogeneity of the experimental platforms used to derive biomarkers (e.g., in vitro, in vivo models, formalin-fixed paraffin-embedded (FFPE) and/or frozen tumor biopsies, plasma/serum, etc.) and technologies employed (qRT-PCR, digital PCR, DNA/RNA-seq, microarray, mass spectrometry etc.), rather than the experimental design (mono/multicentric, cohort/cross-sectional/case–control/case–cohort studies, etc.), perhaps represent the main obstacles toward the standardization of methodologies for optimal biomarker identification and quantification. Recently, research consortia were ad hoc created, to overcome some of these issues and to provide gold standards for biomarker screening and validation, to the research community (see, for example, the miRQC study [19]). It would also be desirable to launch international consortia in order to validate multi-biomarker panels of different origin (e.g., DNA/RNA-based, proteins, lipids, metabolic, etc.) by using shared cohorts of samples, large enough and well-controlled in terms of eligibility criteria, completeness of clinical, and pathological information, and long-term follow-up. This will be paramount to permit a comprehensive analysis of biomarkers’ accuracy and of their correlation with clinical and pathological parameters. An effort toward this direction was recently undertaken by “Biomarkers Consortium” supported by the Foundation for the National Institutes of Health (FNIH) [98]. 

## Figures and Tables

**Table 1:** Performance of various cell-free microRNA (cf-miRNA) biomarkers diagnostic for lung cancer.

Authors	PubMed ID	miRNA (*N*)	AUC	Sample Type	CT
Bianchi et al. [24]	21744498	34	0.89	Serum	*
Montani et al. [14]	25794889	13	0.85	Serum	*
Boeri et al. [25]	21300873	13	0.88	Plasma	*
Sozzi et al. [15]	24419137	24	-	Plasma	*
Wozniak et al. [22]	25965386	24	0.78§	Plasma	
Nadal et al. [26]	26202143	4	0.99	Serum	
Chen et al. [27]	21557218	10	0.97	Serum	
Zhu et al. [28]	27093275	4	0.97†	Serum	
Shen et al. [29]	21864403	3	0.86	Plasma	
Lin et al. [30]	28580707	3	0.87	Plasma	

List of studies reporting the development of cf-miRNA-based biomarkers diagnostic for lung cancer. The number of miRNA (N) in each diagnostic signature is reported together with the performance (AUC, i.e., area under curve) and the type of biospecimen where biomarkers were derived (Serum or Plasma). CT, asterisks indicate studies which performed validation of biomarkers on actual LD-CT screening trials. § Predicted performance when applied to independent samples; † miRNAs combined with carcinoembryonic antigen (CEA); PubMed identifiers (PubMed ID) are reported to allow retreiving cited publications.

**Table 2:** TEX biomarkers diagnostic in lung cancer.

Authors	PubMed ID	Marker Type	Marker (*N*)	Sample Type	Cohort Type (*N*)	AUC	SE	SP
Park et al. [44]	28541032	Raman signals	-	Cell Media	Lung cancer cells and alveolar cells	-	0.95	0.97
Ueda et al. [58]	25167841	CD91 combined with CEA (protein)	2	Serum	Lung cancer patients (165) Interstitial pneumonia patients (29) Healthy donors (64)	0.88	0.71	0.92
Clark et al. [45]	26739763	EGFR, GRB2, and SRC (protein)	3	Cell Media	NSCLC cell lines and human bronchial epithelial cells	-	-	-
Jakobsen et al. [59]	25735706	Protein signature	30	Plasma	ADC stage IIIA–IV (109) Healthy donors (110)	0.83	0.75	0.76
Sandfeld-Paulsen et al. [60]	27343445	Protein signature	10	Plasma	Lung cancer patients (431) Healthy donors (150)	0.74	0.71	0.69
Wang et al. [61]	29573061	LBP (protein)	1	Serum	NSCLC non-metastatic (94) NSCLC metastatic (89) Healthy donors (90)	0.80* 0.68§	0.83* 0.69§	0.67* 0.67§
Li et al. [62]	21557262	LRG1 (protein)	1	Urine	Healthy donors (10) NSCLC non-metastatic (8)	-	-	-
Castellanos-Rizaldos et al. [48]	29535126	EGFR T790M (mutation)	1	Plasma	NSCLC T790M-positive (102) NSCLC T790M-negative (108)	0.96	0.92	0.89
Cazzoli et al. [49]	28789823	miR-200b-5p, miR-378a, miR-139-5p, and miR-379 miR-629, miR-30a-3p, miR-100, miR-200b-5p, miR-154-3p, and miR-151a-5p	4 6	Plasma	NSCLC (20)/Healthy donors (10) Granuloma (30) and ADC (50)	0.91 0.76	0.98 0.96	0.72 0.60
Grimolizzi et al. [51]	29127370	miR-126	1	Plasma	NSCLC (45) Healthy donors (31)	0.86	-	-
Jin et al. [50]	28606918	let-7b-5p, let-7e-5p, miR-23a-3p, and miR-486-5p	4	Plasma	NSCLC stage I (46) Healthy donors (42)	0.90	0.80	0.92
Zhang et al. [52]	28623135	MALAT-1 (lncRNA)	1	Serum	NSCLC (77) Healthy donors (30)	0.70	0.60	0.81

AUC, area under curve; SE, sensitivity; SP, specificity; NSCLC, non-small cell lung cancer; ADC, adenocarcinoma; * metastatic and non-metastatic NSCLC; § Healthy donors and patients with NSCLC; PubMed identifier (PubMed ID) are reported to allow retreiving cited publications.
